# On the size and velocity distribution of cosmic dust particles entering the atmosphere

**DOI:** 10.1002/2015GL065149

**Published:** 2015-08-13

**Authors:** J. D. Carrillo‐Sánchez, J. M. C. Plane, W. Feng, D. Nesvorný, D. Janches

**Affiliations:** ^1^ School of ChemistryUniversity of LeedsLeedsUK; ^2^ National Centre for Atmospheric ScienceUniversity of LeedsLeedsUK; ^3^ Department of Space StudiesSouthwest Research InstituteBoulderColoradoUSA; ^4^ Space Weather LaboratoryGSFC/NASAGreenbeltMarylandUSA

**Keywords:** cosmic dust, comets, meteor radar, meteoric ablation, cosmic spherules, mesospheric metals

## Abstract

The size and velocity distribution of cosmic dust particles entering the Earth's atmosphere is uncertain. Here we show that the relative concentrations of metal atoms in the upper mesosphere, and the surface accretion rate of cosmic spherules, provide sensitive probes of this distribution. Three cosmic dust models are selected as case studies: two are astronomical models, the first constrained by infrared observations of the Zodiacal Dust Cloud and the second by radar observations of meteor head echoes; the third model is based on measurements made with a spaceborne dust detector. For each model, a Monte Carlo sampling method combined with a chemical ablation model is used to predict the ablation rates of Na, K, Fe, Mg, and Ca above 60 km and cosmic spherule production rate. It appears that a significant fraction of the cosmic dust consists of small (<5 µg) and slow (<15 km s^−1^) particles.

## Key Points

- The atmospheric impacts of three cosmic dust models are compared
- Slow cometary particles produce the measured cosmic spherule accretion rate
- These particles also produce significant differential ablation in the mesosphere

## 1 Introduction

Estimates of the global input rate of cosmic dust particles into the Earth's atmosphere vary from ~3 to 300 metric tons per day (t d^−1^) [*Plane*, 2012]. One of the reasons for this 2 order‐of‐magnitude spread is that the entire particle mass distribution, which extends over about 9 orders of magnitude from 10^−10^ to 10^−1^ g (in the range which makes most contribution to the daily input), cannot be measured directly by a single technique [*Plane*, 2012]. Measurements either cover a subset of the mass/velocity distribution or a fraction of the ablation products, e.g., radar observations of meteors [*Janches et al.*, 2014], lidar measurements of the vertical flux of metal atoms in the mesosphere/lower thermosphere (MLT) [*Gardner and Liu*, 2014], and measurements of the surface accumulation flux of cosmic spherules [*Taylor et al.*, 1998] and meteoric smoke particles [*Dhomse et al.*, 2013].

A less direct estimate of the ablation flux has been provided by global modeling of the observed Na, Fe, and Mg atom concentrations in the MLT [*Feng et al.*, 2013; *Marsh et al.*, 2013; *Langowski et al.*, 2015]. These studies have revealed an important problem: whereas the CI (Carbonaceous Ivuna) chondritic ratios of Na:Fe:Mg are 1:15:17 [*Asplund et al.*, 2009], global models require relative ablation rates of 1:4:1. Even more striking is that atomic Ca, which has a similar chondritic abundance to Na, is depleted in the upper mesosphere by factors of 50–100 [*Plane et al.*, 2015].

In this paper we consider three quite different models of the cosmic dust mass/velocity distribution: an astronomical model constrained by observations of IR emission from the Zodiacal Dust Cloud [*Nesvorný et al.*, 2010, 2011], a model derived from measurements on a spaceborne dust detector [*Love and Brownlee*, 1993; *McBride et al.*, 1999], and an astronomical model describing the portion of the incoming flux measured by meteor head echo detections with high‐power and large‐aperture (HPLA) radars [*Fentzke and Janches*, 2008; *Pifko et al.*, 2013]. The Meteoric Input Function (MIF), which defines the injection rate of each meteoric element as a function of time and location [*Feng et al.*, 2013], is derived for each of these models by processing the distribution through a meteoric chemical ablation model [*Vondrak et al.*, 2008]. The relative injection rates of the different metals are then compared with those required to model the observed relative abundances of the mesospheric metals. The predicted surface accretion rate of cosmic spherules (i.e., cosmic dust particles which melt but do not completely ablate in the upper atmosphere) is also compared with the measured accretion rate at the bottom of an ice chamber at the Amundsen‐Scott base at South Pole [*Taylor et al.*, 1998, 2007] and in the Greenland ice cap [*Maurette et al.*, 1987].

## 2 Models of Cosmic Dust in the Near‐Earth Environment

The three models used as case studies have completely different mass and/or velocity distributions. First is the Zodiacal Dust Cloud model developed by *Nesvorný et al.* [2010]. The zodiacal cloud is a circumsolar disk formed by small debris particles produced by comets and asteroidal collisions. In the model, submillimeter particles from these sources are launched and tracked as their orbits evolve under the influence of solar radiation pressure, Poynting‐Robertson drag, and planetary perturbations. Comparison with observations of infrared emission from the Zodiacal Cloud observed by the Infrared Astronomical Satellite (IRAS) indicates that the majority (>80%) of the IR emission is produced by particles originating from Jupiter Family Comets (JFCs) [*Nesvorný et al.*, 2010, 2011]. These should represent between 50 and 70% of the incoming flux. This model is termed here the z‐MIF.

The second model—the d‐MIF—is derived from analysis of small particle impact craters on the aluminum and gold panels which were located on the Long Duration Exposure Facility (LDEF) [*Love and Brownlee*, 1993], which was exposed in near‐Earth orbit to particle impacts for 5.8 years. The diameter and depth of an impact crater depends largely on the velocity of the impacting particle, and the geometry and physical properties (density, tensile strength, and hardness) of the particle and target [*McBride et al.*, 1995]. The mass distribution was then derived by assuming a constant impact velocity of 16.9 km s^−1^ [*Love and Brownlee*, 1993], although the distribution is sensitive to the assumed velocity distribution [*Taylor*, 1995; *Mathews et al.*, 2001].

The third model—the r‐MIF—was developed to interpret meteor head echo observations made by high‐powered large‐aperture (HPLA) radars, in terms of the extraterrestrial sporadic meteoroid apparent sources [*Fentzke and Janches*, 2008]. This model uses the global mass flux reported by *Ceplecha et al.* [1998], together with current knowledge of the velocity and radiant distributions of these sources, to explain the diurnal, seasonal, and geographical variability of the HPLA observations. The model is constrained with radar systems that have different detection sensitivities to the different incoming meteor populations [*Janches et al.*, 2008; *Fentzke et al.*, 2009; *Pifko et al.*, 2013]. The model shows that although the Earth's apex‐centered radiant source, which is characterized by high geocentric speeds (~55 km s^−1^), appears to be ~33% of the meteoroids in the solar system at 1 AU, it accounts for the majority of the HPLA radar detections. The remaining observed meteors originate mostly from the helion and antihelion sources, which seem to be the majority of the incoming particles (~50%), with a very small, but diurnally constant, contribution from the south and north toroidal sources.

**Figure 1:**
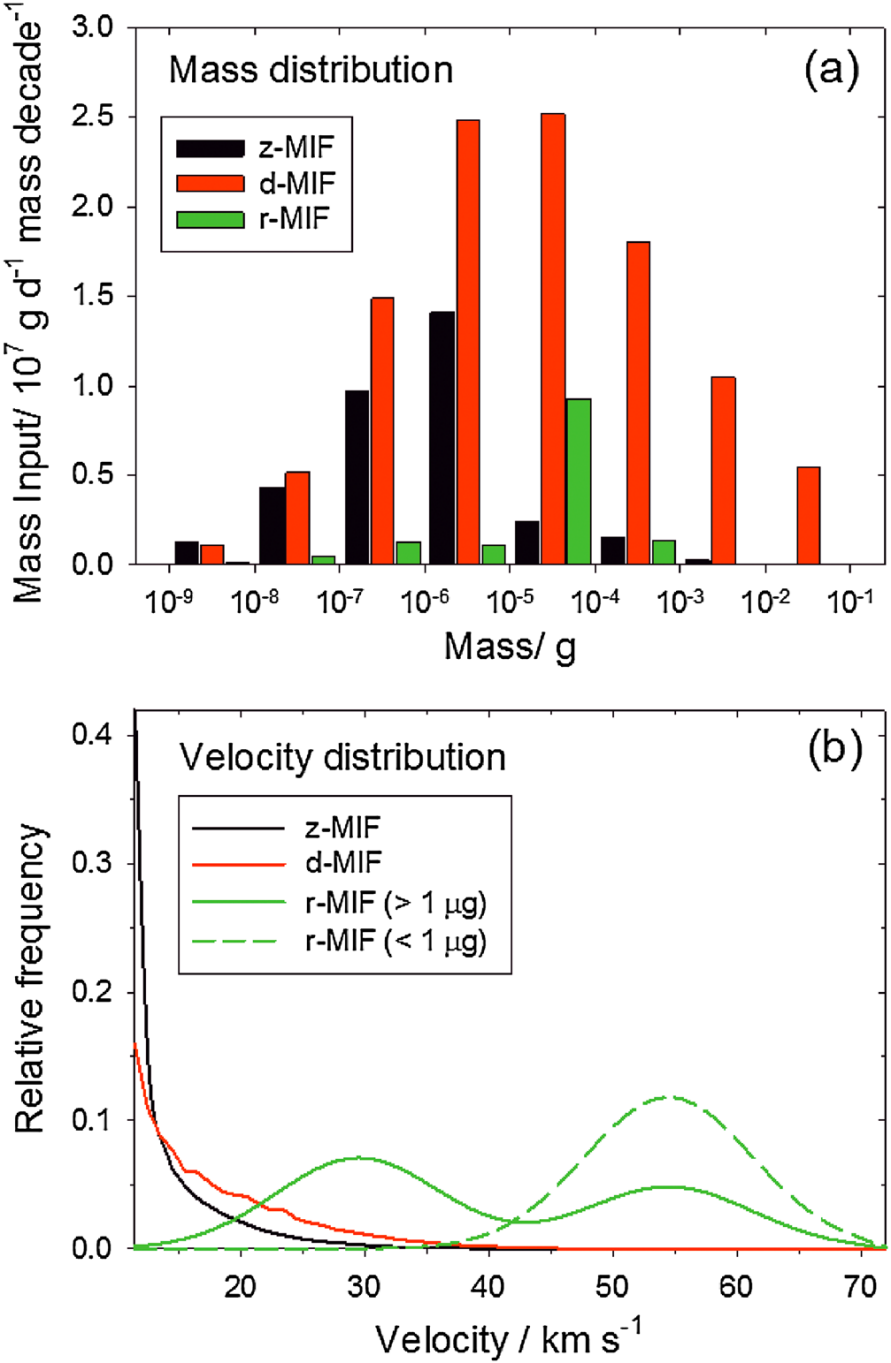
(a) Histogram of the particle mass distributions and (b) entry velocity distributions for the z‐MIF (black), d‐MIF (red), and r‐MIF (green) models.

Figure 1a shows a histogram of the particle mass distributions for each of the three models. The mass distribution is expressed in terms of mass flux per decade versus the mass range from 10^−9^ to 0.1 g, which covers the bulk of the incoming daily material [*Ceplecha et al.*, 1998]. In the d‐MIF distribution, the median input mass of the incoming dust particles is ~10 µg, with a total input rate of 110 ± 55 t d^−1^ [*Love and Brownlee*, 1993]. For the r‐MIF and z‐MIF models, the mass distribution is shifted to smaller mass ranges with a median input mass of ~1 µg. The total input rate in the z‐MIF model is 34 ± 17 t d^−1^, although there may be 30–50% of additional mass input from asteroids and long‐period comets [*Nesvorný et al.*, 2011]. The r‐MIF predicts an input rate of 14 ± 3 t d^−1^ [*Fentzke and Janches*, 2008; *Janches et al.*, 2014].

Figure 1b shows the entry velocity distributions of the three models. The velocity ranges from 11.5 km s^−1^ for particles in a prograde orbit to 72.5 km s^−1^ for those in a retrograde orbit. The z‐MIF velocity distribution is sharply peaked to low velocities (average = 14 km s^−1^), since the majority of particles are predicted to be in near‐prograde orbits originating from the helion and antihelion sporadic sources [*Nesvorný et al.*, 2011].

In contrast, the r‐MIF velocity distribution depends strongly on the mass range. For masses ≥ 0.1 µg, the velocity distribution follows a bimodal trend where the dominant peak is located at 30 km s^−1^ and there is a secondary maximum at 55 km s^−1^. Meanwhile, there is a single peak at 55 km s^−1^ for masses < 0.1 µg, because the r‐MIF takes into account only the portion of the incoming flux that is detectable by the radar [*Fentzke and Janches*, 2008]. In the case of the d‐MIF, the LDEF velocity distribution (Figure 1b) is taken from *McBride et al.* [1999] and has an average of 18 km s^−1^.

## 3 Modeling Ablation and Cosmic Spherule Production

We employ here the Chemical ABlation MODel (CABMOD) [*Vondrak et al.*, 2008] to predict the fate of each meteoroid with specified mass, velocity, and entry angle after it enters the atmosphere at 500 km altitude. In addition to a standard treatment of meteor physics—the balance of frictional heating by radiative losses and by the absorption of heat energy through temperature increases, melting, phase transitions, and vaporization—CABMOD includes sputtering of elemental constituents by inelastic collisions with air molecules before the meteoroid melts, followed by evaporation of atoms and oxides from the molten particle if its temperature exceeds the melting point above 1800 K. Note that the term ablation covers both sputtering and evaporation from the melt. We assume the particles have an ordinary chondrite composition (essentially MgFeSiO_4_ with small amounts of other metal oxides). This is supported by the analysis of Comet Wild dust samples [*Gainsforth et al.*, 2015] and the observation that S‐type asteroids, the probable parent bodies of ordinary chondrites, are the dominant group between 1 and 2.4 AU [*McSween*, 1999]. The particles are also assumed to be fully mixed with a particle density of 2 g cm^−3^ [*Vondrak et al.*, 2008].

CABMOD predicts the ablation rate profiles of Na, K, Fe, Mg, Si, Ca, Al, and Ti. If the meteoroid has not ablated completely, then the model determines whether the particle melted at any point along the trajectory and thus became a cosmic spherule, or survived entry unchanged to become an unmelted micrometeorite. Complete melting of the particle, and hence formation of a spherule if only partial evaporation of the particle subsequently occurs, is assumed to occur if the meteoroid temperature reaches 1800 K [*Vondrak et al.*, 2008]. Solidified spherules are denser than cosmic dust particles; here we use a mean density of 3.2 g cm^−3^ [*Kohout et al.*, 2014] to estimate the spherule size for comparison with measurements. Figure S1 in the supporting information is a flow chart with accompanying text which explains in more detail how CABMOD operates.

For simplicity, CABMOD was run with a constant atmospheric density profile (March, 40°N). Each meteoroid in the r‐MIF and z‐MIF models has a specified mass, velocity, and entry angle. These models contain 2.7 × 10^7^ and 6.7 × 10^6^ individual cosmic dust particles, respectively. In view of these very large numbers, the following procedure was adopted to integrate efficiently across the mass/velocity/entry angle distributions. Each mass decade in the distribution was divided into five bins. A Monte Carlo procedure was used to sample the particle velocity and entry angle distributions of particles within each bin, and the resulting elemental ablation profiles and residual particle masses coadded. The results for each bin were then summed to yield the integrated ablation profiles.

In order to determine the minimum number of particles that should be sampled in each bin, the ratios of the integrated ablation profiles for each metal relative to Na were compared for different sample sizes. No significant improvement was observed when increasing the sample size above 200. For instance, for the z‐MIF model, the ablation ratios for a sample size of 200 were Fe:Na = 5.9 ± 0.2, Mg:Na = 4.0 ± 0.2, and Ca:Na = (6.1 ± 0.5) × 10^−2^, compared with Fe:Na = 5.9 ± 0.1, Mg:Na = 4.0 ± 0.1, and Ca:Na = (6.2 ± 0.3) × 10^−2^ for a sample size of 500. The total numbers of particles sampled were then 9500, 8600, and 11,200 for the z‐MIF, r‐MIF, and d‐MIF, respectively. In the case of the d‐MIF model where the entry angle is not specified, a constant value of 35° was used since the integrated ablation rates are relatively insensitive to this parameter [*Vondrak et al.*, 2008].

## 4 Results and Discussion

### 4.1 Differential Ablation

**Figure 2:**
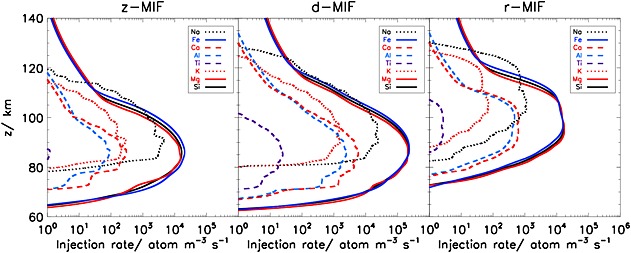
Ablation rate profiles for individual elements, integrated over the available mass ranges of the z‐MIF, d‐MIF, and the r‐MIF models. The meteoroid mass covers the range 10^−9^ to 10^−3^ g (z‐MIF and r‐MIF) and 10^−14^ to 10^−3^ g (d‐MIF).

The elemental ablation rate profiles for the three models are plotted in the panels of Figure 2. These profiles are integrated over the meteoroid velocity, zenith angle (in the case of the z‐MIF and r‐MIF), and mass distributions to yield the total ablation rates of the individual elements. In all cases, the most volatile elements (Na and K) ablate 10–15 km higher than the main constituent elements (Fe, Mg, and Si), which in turn ablate a few kilometers higher than the most refractory elements (Ca, Al, and Ti). As expected, the ablation profiles for the r‐MIF model are 10–20 km higher than the corresponding profiles for the z‐MIF and d‐MIF models because of the much faster velocity distribution (Figure 1b). As a result, sputtering is also more important in the r‐MIF model. Note that although the velocity distribution for the d‐MIF model is shifted to somewhat higher velocities than the z‐MIF (Figure 1b), the larger particles in the d‐MIF distribution (Figure 1a) take longer to reach melting point with the result that ablation persists to lower altitudes than in the z‐MIF.

Table 1 lists the global mass balance for each model: that is, how the incoming mass is partitioned between unmelted micrometeorites, cosmic spherules, and the ablated mass. The ablated mass is then broken down by element (the percentage of each element which ablates from the incoming total is also listed). The z‐MIF and the r‐MIF models are at opposite extremes: 91% of the total mass ablates in the r‐MIF model compared with only 12% of the mass in the z‐MIF. This significant difference largely arises because the velocity distribution of the fast velocity distribution of the r‐MIF.

**Table 1:** Global Mass Balance of the z‐MIF, d‐MIF, and r‐MIF Models^a^

Mass Flux	z‐MIF (t d^−1^)	d‐MIF (t d^−1^)	r‐MIF (t d^−1^)
Unmelted micrometeorites	22.0	23.2	0.3
Cosmic spherules	8.1	35.4	1.2
Ablated atoms	3.9	51.4	12.5
*Na*	*0.1 (40%)*	*0.7 (83%)*	*0.1 (95%)*
*K*	*0.01 (36%)*	*0.07 (76%)*	*0.01 (86%)*
*Fe*	*1.5 (16%)*	*17.2 (56%)*	*3.7 (94%)*
*Si*	*0.6 (11%)*	*7.9 (45%)*	*2.0 (90%)*
*Mg*	*0.4 (8%)*	*6.6 (41%)*	*1.8 (88%)*
*Ca*	*0.01 (2%)*	*0.3 (20%)*	*0.1 (52%)*
*Al*	*2.4 · 10^−3^ (0.5%)*	*0.08 (6%)*	*0.05 (28%)*
*Ti*	*5.4 · 10^−5^ (2% )*	*1.4 · 10^−3^ (18%)*	*6.5 · 10^−4^ (65%)*
*O*	*1.3 (10%)*	*18.5 (45%)*	*4.6 (87%)*
Total	34	110	14

a Note that the mass flux of ablated atoms is broken down by element in the italicized entries, where the number in parenthesis shows the percentage fraction of each element that ablates from its total atmospheric input.

The differences between the models are even more dramatic when considering the comparative ablation rates of individual elements. Starting with Na, which is a relatively volatile metal and therefore ablates efficiently, 40% of the total incoming Na ablates in the case of the z‐MIF model compared with 95% for the r‐MIF. In fact, the Na ablation rates are almost the same for both models (0.1 t d^−1^), because the smaller total mass input of the r‐MIF is compensated by the higher ablation fraction of Na. For more refractory metals, the dust velocity distribution becomes more critical. Taking Ca as an extreme case, only 2% of the incoming Ca ablates for the z‐MIF, compared with 52% for the r‐MIF. Differential ablation is defined as a departure from the chondritic ratio of two elements. The chondritic Na:Ca ratio is 0.96 [*Asplund et al.*, 2009]. For the r‐MIF, the Na:Ca ratio increases slightly to 1.5. In contrast, the z‐MIF exhibits pronounced differential ablation with a Na:Ca ratio of 16.1.

The velocity distribution of the d‐MIF is only slightly faster than the z‐MIF (Figure 1b). Thus, similar ablation behavior might be expected. However, the d‐MIF mass distribution is shifted to much heavier particles (Figure 1a), which exhibit less differential ablation because they reach higher temperatures during atmospheric entry, so that a higher fraction of the refractory elements ablate [*Vondrak et al.*, 2008, Figure 12]. Thus, the Na:Ca ratio for the d‐MIF model is only 4.6.

**Figure 3:**
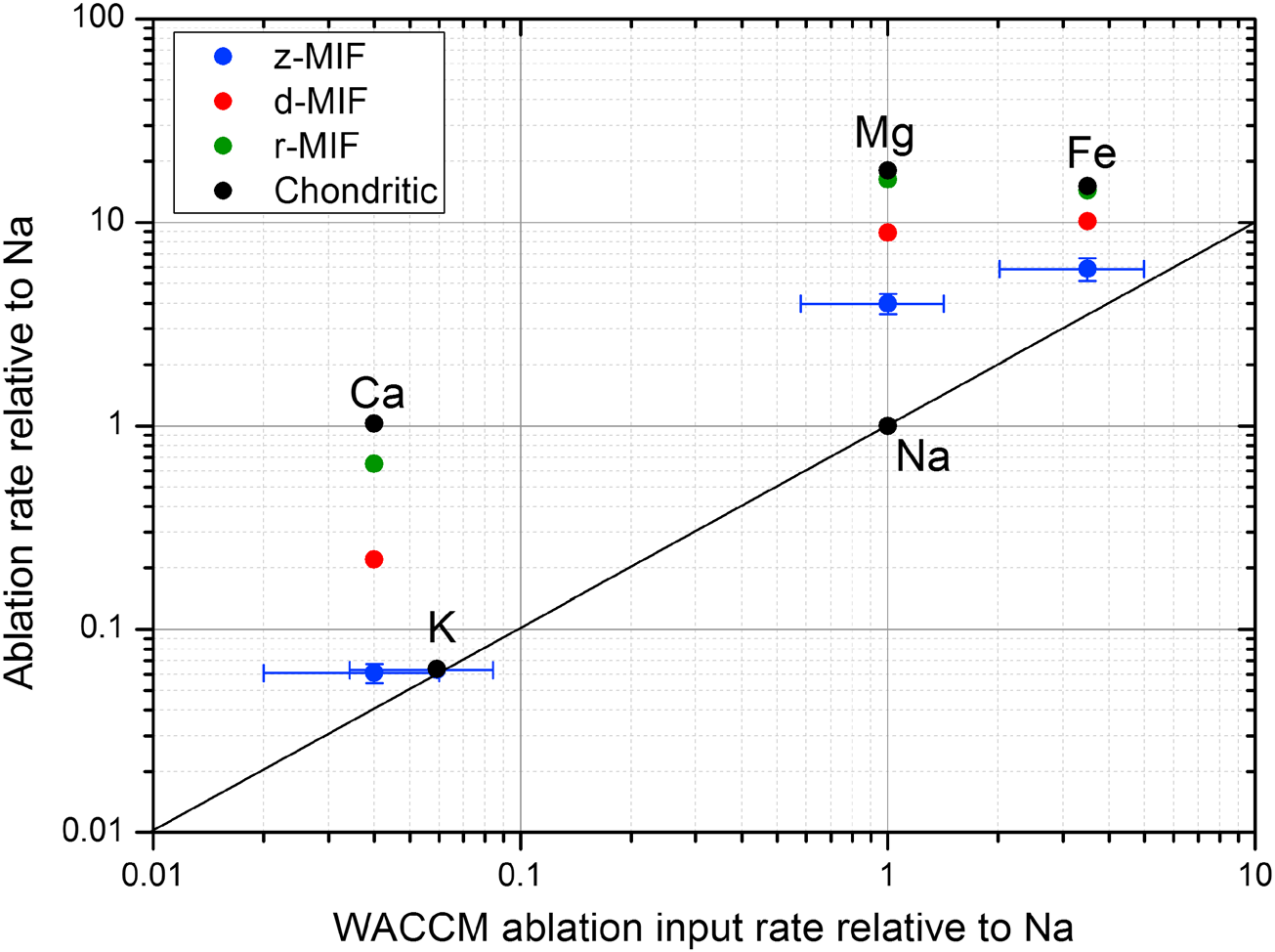
Ablation rates for Fe, Ca, Mg, and K relative to Na, produced by the z‐MIF, d‐MIF, and r‐MIF models, plotted against the relative input rates required to model the global metal atom layers in the MLT. The solid line is the 1:1 correlation line. The error bars on the z‐MIF points indicate the uncertainty in the measured ratios (horizontal) and the cosmic dust melting point (vertical).

The ablation ratios of Fe, Ca, Mg, and K relative to Na are shown in Figure 3 for the three models. The abscissa is the ablation ratio required to match the modeled mesospheric metal layers, within the framework of the Whole Atmosphere Community Climate Model (WACCM), against observations by lidar and satellite [*Feng et al.*, 2013; *Marsh et al.*, 2013; *Plane et al.*, 2014; *Langowski et al.*, 2015]. The ordinate axis represents the ablation ratios from Table 1. The black points show the relative chondritic ratios used in the CABMOD model [*Vondrak et al.*, 2008] and so illustrate the ratios corresponding to an absence of differential ablation. Because Na ablates very efficiently, differential ablation of other elements leads to points vertically below the black points on the plot. The further the points lie above the line of 1:1 correspondence, then the smaller the degree of differential ablation that the cosmic dust model is producing. Inspection of Figure 3 shows that Na and K ablate essentially in their chondritic ratio; hence, the points for all three models lie on top of each other. In contrast, as the elements become more refractory, a larger degree of differential ablation is exhibited. The r‐MIF produces very little differential ablation for Fe, Mg, or Ca, whereas the z‐MIF produces a Ca:Na ratio that is close to that required by WACCM. However, even the z‐MIF does not produce sufficient differential ablation of Mg and Fe, one possible explanation for this is that Na is enriched in cometary particles, compared to the CI ratio. Indeed, Na enrichments in cometary particles have been reported recently [*Schulz et al.*, 2014; *Gainsforth et al.*, 2015].

### 4.2 Accretion of Cosmic Spherules

A collection of thousands of well‐preserved cosmic spherules from the bottom of an ice chamber at the South Pole was used to estimate the global flux of 50–700 µm diameter cosmic spherules to be 4.4 ± 0.8 t d^−1^ [*Taylor et al.*, 1998, 2007]. The flux of spherules in this size range for the z‐MIF model is 6.8 ± 3.4 t d^−1^, which is in good agreement with the South Pole measurement. In contrast, the spherule flux is only 0.5 ± 0.1 t d^−1^ for the r‐MIF, and the d‐MIF model produces a much higher flux of 29.7 ± 14.9 t d^−1^. The spherule flux estimated from the z‐MIF model also falls within the range of 1.4–19.2 t d^−1^ from the deep‐sea sediment record [*Peng and Lui*, 1989]. Lastly, *Maurette et al.* [1987] reported a spherule flux of 6.0 t d^−1^ within the size range 50–300 µm, from micrometeorites collected in the Greenland ice cap. The z‐MIF model again provides the best agreement with a flux in this size range of 6.2 ± 3.1 t d^−1^, compared with 0.4 ± 0.1 and 25.9 ± 13.0 t d^−1^ for the r‐MIF and d‐MIF models, respectively.

### 4.3 Input Fluxes of Na and Fe

We now compare the *absolute* ablation fluxes of Na and Fe to observations and models. Na and Fe resonance wind‐temperature lidars have recently been used to measure the vertical fluxes of these metals in the MLT. Gardner and coworkers have reported two estimates for the global Na input flux of 0.28 ± 0.05 t d^−1^ [*Gardner et al.*, 2014] and 0.30 ± 0.05 t d^−1^ [*Huang et al.*, 2015], which are a factor of 2.8–3.0 times higher than the z‐MIF model. In the case of Fe, the lidar‐based estimate of 4.29 ± 0.75 t d^−1^ [*Huang et al.*, 2015] is a similar factor of 2.9 times larger than the z‐MIF.

Given that the stated uncertainty in the z‐MIF is a factor of 2 [*Nesvorný et al.*, 2011], this discrepancy may not be as significant as it appears. Moreover, HPLA radars mostly observe a different group of relatively fast particles, as evidenced by the completely different velocity distributions in Figure 1b [*Janches et al.*, 2014]. Thus, to a first approximation, the r‐MIF can be added to the z‐MIF, yielding (from Table 1) Na and Fe fluxes of 0.2 and 5.2 t d^−1^, which are reasonably close to the lidar‐based estimates. Interestingly, this Fe flux is in sensible accord with the accretion rate of meteoric smoke particles in polar ice, which indicates a global Fe ablation flux of around 8 ± 4 t d^−1^ [*Dhomse et al.*, 2013]. However, it should be noted that although the cosmic spherule flux would be little altered by adding the two MIFs together (since the r‐MIF produces relatively few spherules—Table 1), the degree of differential ablation for Ca, Mg, and Fe would be worse than for the z‐MIF alone.

### 4.4 Model Uncertainties

One parameter in CABMOD to which there is significant sensitivity is the melting point of the particle [*Vondrak et al.*, 2008]. This is set to 1800 K, which is typical for olivine Mg_2*x*_Fe_2(1 − *x*)_SiO_4_ of composition *x* = 0.5 (that is, a Mg/Fe ratio ~1, as found in both S‐type asteroidal and cometary particles—see above). We have rerun the model with the melting point varied from 1700 K (*x* = 0.25) to 1900 K (*x* = 0.75). For the z‐MIF, which is most sensitive to this parameter, the cosmic spherule production rate decreases from 9.4 to 6.9 t d^−1^, which is only ± 16% from the standard model. The effect on the ablation ratios is also small (Figure 3). For example, the Fe:Na ratio increases from 6.13 to 7.30 and the Ca:Na ratio from 0.06 to 0.07.

The metal atom injection rates in WACCM are optimized to yield the best fits to metal layer observations [*Feng et al.*, 2013; *Marsh et al.*, 2013; *Langowski et al.*, 2015]. The uncertainty in the absolute metal atom concentration measured by lidar is typically ±30%, similar to satellite measurements of Mg [*Langowski et al.*, 2015]. The uncertainty in the measured metal atom:Na ratio is thus ±42%. In WACCM, the transport (residual circulation and diffusion) and concentration fields of the neutrals (O, H, O_3_, etc.) and charged species (NO^+^, O_2_^+^, and electrons) affect all metal species in the same way and should not contribute to the uncertainty in the metal atom ratios. Of course, there is uncertainty in the metal chemistry, both in individual rate coefficients (~30 reactions per metal [*Plane et al.*, 2015]) and the possibility of unknown reactions. However, a fairly strict test of the completeness of the chemistry of a particular metal is that the modeled metal atom layer satisfactorily reproduces the peak height, width, top and bottom scale heights, and diurnal/seasonal variations [*Plane et al.*, 2015]. Thus, the additional uncertainty in the chemistry is likely to be comparatively small, and so the uncertainties in the metal:Na ablation ratios required by WACCM (abscissa in Figure 3) are probably no more than ±60% (shown for the z‐MIF in Figure 3).

## 5 Conclusions

This study shows that a significant fraction of the cosmic dust entering the Earth's atmosphere needs to consist of small (<5 µg) and slow (<15 km s^−1^) particles in order to explain the measured accretion rate of cosmic spherules at the surface, as well as the significant differential ablation of the more refractory meteoric metals with respect to Na in the MLT. Of the three MIFs selected for this study, the Zodiacal Dust Cloud model (z‐MIF) seems to do best when judged against these criteria.

However, there are at least two unresolved issues. First, *Janches et al.* [2014] have shown that the z‐MIF predicts a flux of relatively fast particles (>15 km s^−1^) which are not observed by HPLA radars; this suggests that further refinements to the JFC component of the z‐MIF are required, even before other components (asteroidal, long‐period comets) are added. Second, the metal ablation rates required to model the Fe and Na layers in WACCM [*Feng et al.*, 2013; *Marsh et al.*, 2013] are factors of 3–5 times smaller than the z‐MIF (Table 1) and 10–14 times smaller than lidar‐based estimates [*Huang et al.*, 2015]. One implication is that additional vertical transport in the upper mesosphere is required to accommodate increased metal ablation rates and produce metal layers which still matched observations. This would have wider implications in the increased downward transport of heat and chemical constituents such as atomic O and NO.

## Erratum

In Figure 1(a) in this paper, the x axis of the graph was wrongly plotted (a factor of 10 had been included erroneously in the plotting software). The figure has since been corrected and this version may be considered the authoritative version of record.

## Supporting information



Supporting Information S1
