# Supplementary material for: On the size and velocity distribution of cosmic dust particles entering the atmosphere
Source: Geophys Res Lett. 2015 Aug 13;42(15):6518–25. doi: 10.1002/2015GL065149 (PMC4950038; doi:10.1002/2015GL065149)
Supplement: Supplementary file 1 — Supporting Information S1 [file GRL-42-6518-s001.doc]

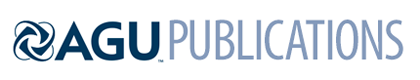


*Geophysical Research Letters*

Supporting Information for

**On the size and velocity distribution of cosmic dust particles entering the atmosphere**

J. D. Carrillo-Sánchez^1^, J. M. C. Plane^1^, W. Feng^1,2^, D. Nesvorný^3^, D. Janches^4^

^1^School of Chemistry, University of Leeds, Leeds LS2 9JT, UK, ^2^ National Centre of Atmospheric Science, University of Leeds, Leeds, LS2 9JT, UK, ^3^ Department of Space Studies, SouthWest Research Institute, 1050 Walnut St., Suite 30, Boulder, CO 80302, ^4^ Space Weather Lab., Mail Code 674, GSFC/NASA, Greenbelt, MD 20771

**Contents of this file**

Figure S1

**Introduction**

The purpose of this Supporting Information is to describe in greater detail how the cosmic spherule flux and the elemental ablation profiles are determined from a given Meteoric Input Function (MIF). The MIF provides distributions of dust particle mass and velocity (and entry angle for the z-MIF and r-MIF). Figure S1 is a flow-chart illustrating how the Chemical Ablation Model is used to determine the outcome for an individual cosmic dust particle entering the Earth’s atmosphere. As described in Section 3 of the main paper, each mass decade in the dust mass distribution is divided into 5 bins. For a selected bin mass, a Monte Carlo procedure is used to sample the particle velocity and entry angle distributions. The particle is then launched into the atmosphere with a specified mass (i.e. the selected mass bin), velocity and entry angle, starting from an altitude of 500 km.

Initially, the particle loses mass through sputtering with air molecules and starts to heat. At each time step the model tests to see whether the particle has reached a specified melting point (default = 1850 K). The onset of melting is actually described using a sigmoidal function with the melting point at the mid-point, as described in . Once the particle has melted, the thermodynamic code MAGMA is called to determine the equilibrium vapor pressures of the constituent atoms and oxides above the melt. These vapor pressures are used to determine the evaporative mass loss rate, assuming Langmuir evaporation through the Herz-Knudsen relation . Mass loss through sputtering also continues, though this is orders of magnitude lower than evaporative loss once the particle has melted. The particle continues to be heated through collisions with atmospheric molecules, which is offset by radiative cooling and evaporation.

Evaporation also changes the particle composition since elements evaporate at different rates (initially just the relatively volatile elements Na and K, followed by Fe, Mg and Si, and finally the most refractory species Ca, Al and Ti). After each time step, the new composition of the molten particle is computed. If the particle temperature remains above the melting point, the MAGMA routine is called to compute the new elemental vapor pressures and evaporation continues. If, however, the particle has cooled below its melting point, or has completely evaporated (mass has fallen below a threshold value of 10^-10^ g), then CABMOD is exited and the fate of the particle assessed: if the particle did not reach its melting point during entry, then it produced an unmelted micrometeorite; if the particle melted but did not completely ablate, then it formed a cosmic spherule.

The cycle is then repeated for typically 200 cosmic dust particles in each mass bin (with the initial velocity and entry angle chosen pseudo randomly). The resulting elemental ablation profiles and residual unmelted micrometeorite and spherule masses are co-added. Finally, the results for each bin are weighted according to the mass distribution of the MIF, and then summed to yield the integrated ablation profiles and total mass of residual particles.

**References**

Schaefer, L., and B. Fegley (2005), Application of an equilibrium vaporization model to the ablation of chondritic and achondritic meteoroids, *Earth Moon Planets*, *95*(1-4), 413-423.

Vondrak, T., J. M. C. Plane, S. Broadley, and D. Janches (2008), A chemical model of meteoric ablation, *Atmos. Chem. Phys.*, *8*, 7015-7031.

**
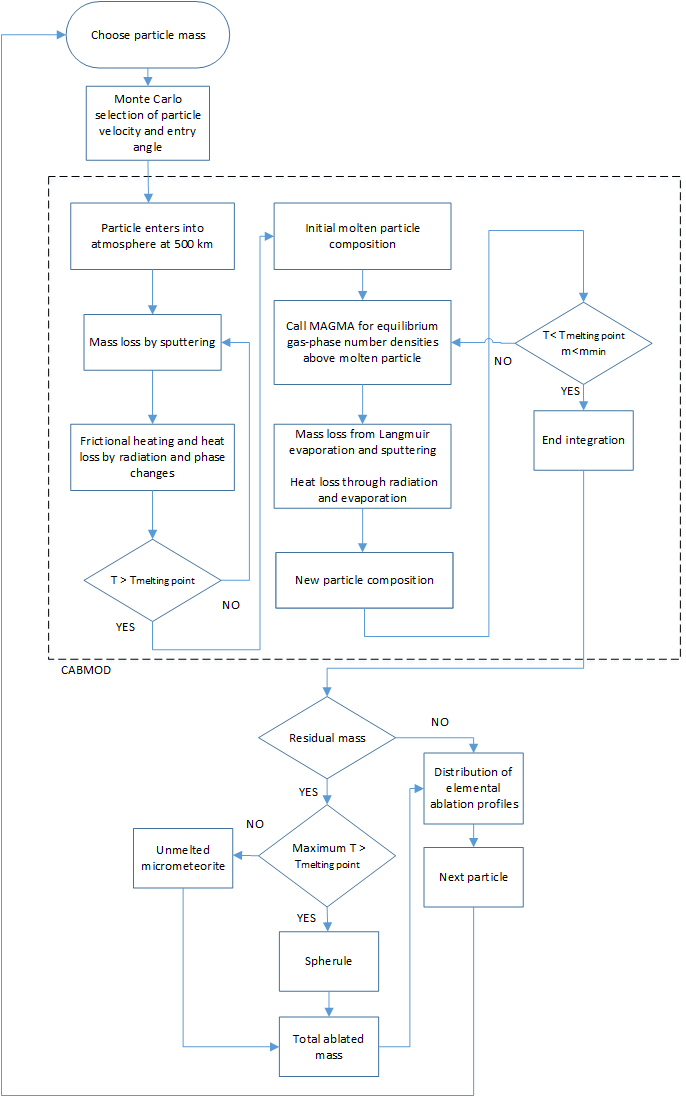
**

**Figure S1.** Flow chart illustrating how the Chemical Ablation Model (CABMOD) determines the ablative mass loss and the residual melted/unmelted mass for a cosmic dust particle entering the atmosphere with a specified initial mass, velocity and entry angle.
